# Naja naja atra venom ameliorates pulmonary fibrosis by inhibiting inflammatory response and oxidative stress

**DOI:** 10.1186/1472-6882-14-461

**Published:** 2014-12-02

**Authors:** Kui Cui, Jian-Qun Kou, Jin-Hua Gu, Rong Han, Guanghui Wang, Xuechu Zhen, Zheng-Hong Qin

**Affiliations:** Department of Pharmacology and Laboratory of Aging and Nervous Diseases, Jiangsu Key Laboratory of Translational Research and Therapy for Neuro-Psycho-Diseases, Soochow University School of Pharmaceutical Science, Suzhou, 215123 China; Department of Pharmacology and Laboratory of Molecular Neuropathology, Soochow University School of Pharmaceutical Science, Suzhou, 215006 China; Department of Pharmacology and Laboratory of Neuropsychopharmacology, Soochow University School of Pharmaceutical Science, Suzhou, 215006 China

**Keywords:** Naja naja atra venom, Pulmonary fibrosis, Hydroxyproline, NF-κB, TGF-βm, Oxidative stress

## Abstract

**Background:**

Naja naja atra venom (NNAV) displays diverse pharmacological actions including analgesia, anti-inflammation and immune regulation.

In this study, we investigated the effects of NNAV on pulmonary fibrosis and its mechanisms of action.

**Methods:**

To determine if Naja naja atra venom (NNAV) can produce beneficial effects on pulmonary fibrosis, two marine models of pulmonary fibrosis were produced with bleomycin (BLM) and lipopolysaccharide (LPS). NNAV (30, 90, 270 μg/kg) was orally administered once a day started five days before BLM and LPS until to the end of experiment. The effects of NNAV treatment on pulmonary injury were evaluated with arterial blood gas analysis, hydroxyproline (HYP) content assessment and HE/Masson staining. The effects of NNAV treatment on inflammatory related cytokines, fibrosis related TGF-β/Smad signaling pathway and oxidative stress were examined.

**Results:**

The results showed that NNAV improved the lung gas-exchange function and attenuated the fibrotic lesions in lung. NNAV decreased IL-1β and TNF-α levels in serum in both pulmonary fibrosis models. NNAV inhibited the activation of NF-κB in LPS-induced and TGF-β/Smad pathway in BLM-induced pulmonary fibrosis. Additionally, NNAV also increased the levels of SOD and GSH and reduced the levels of MDA in BLM-induced pulmonary fibrosis model.

**Conclusions:**

The present study indicates that NNAV attenuates LPS- and BLM-induced lung fibrosis. Its mechanisms of action are associated with inhibiting inflammatory response and oxidative stress. The study suggests that NNAV might be a potential therapeutic drug for treatment of pulmonary fibrosis.

## Background

Pulmonary fibrosis is a progressive and lethal lung disease characterized by accumulation of extracellular matrix and loss of pulmonary function [1]. The disease can be idiopathic or developed as a complication of many respiratory and systemic diseases. Although the etiology of pulmonary fibrosis has not as yet been clearly elucidated, some putative mechanisms involved in the pathogenesis including inflammation and oxidative stress have been intensively explored [2, 3]. Inflammation is the initial response following lung injury. Once activated, inflammatory cells such as neutrophils and macrophages accumulate in the lower airways and consequently release harmful amounts of reactive oxygen species and some pro-inflammatory cytokines and growth factors that regulate the proliferation and secretary activity of alveolar fibroblasts in alveolar wall. The activated fibroblasts produce increasing amounts of matrix proteins, which distort the normal lung architecture and affect gas exchange. Therefore, inhibition of inflammation and oxidative stress represents possible therapeutic strategies [4–8]. Actually, the present treatment recommendations for lung fibrosis are corticosteroid combined with immunosuppressants, anti-inflammatory and anti-oxidant drugs. Unfortunately, the therapeutic effects of these treatments are limited, nonspecific, and largely ineffective [9]. Thus the development of novel agents to ameliorate pulmonary fibrosis is urgently needed.

Snake venoms, as complementary and alternative medicine, display diverse pharmacological effects due to their complex compositions including toxins, enzymes and other bioactive factors. Naja naja atra (Chinese cobra) and its toxic venoms are considered as a medicine in Chinese folk medicine. Our recent research showed that Naja naja atra venom (NNAV) or some specific component of the crude venom (cobrotoxin) significantly suppressed inflammation and rheumatoid arthritis in animal models [10, 11]. Some investigators also reported certain fractions isolated from NNAV have a remarkable free radical scavenging activity in mice [12].

In the light of the association of pulmonary fibrosis pathogenesis with inflammation and cytokines, we speculated that NNAV might have therapeutic effects in lung fibrosis. To test this possibility, we applied two murine pulmonary fibrosis models. One was inflammation-related fibrosis mouse model induced by lipopolysaccharide (LPS), and another was bleomycin (BLM)-induced rat pulmonary fibrosis. On the basis of our previous report, in this study NNAV was reversible denatured by heat and administered orally [10].

## Methods

### Animals

One hundred Kunming (KM) mice (half male and half female, 18–20 g) and 60 male Sprague–Dawley rats (180-220 g) were obtained from the Shanghai SLAC Laboratory Animal Co. Ltd. All animals were housed in a controlled ambient temperature (22±2°C), humidity (40%-70%), a 12 h light/dark cycle with free access to food and water. Animals were acclimated to the housing conditions for 2–3 days before experiments. All experiments and surgical procedures were approved by the Animal Care and Use Committee of Soochow University, which complies with the National Institute of Health Guide for the Care and Use of Laboratory Animals.

### Reagents and drugs

Naja naja atra venom, purchased from Rainbow Snake Farm (Yu Jiang, Jiangxi Province, China), which is certified for having standard biological activities and compositions. NNAV contains neurotoxin 8%-10%, cardiotoxin 35%–40%, nerve growth factor 1%-2%, phospholipase A2 8%, and cobra venom factor (CVF). The doses of NNAV were decided with our previous studies [13]. It was dissolved in sterile saline and heated to 100°C for 10 min, then placed at room temperature (RT) for renaturation and stored at 4°C until use [10]. Both LPS (Sigma-Aldrich Co., USA) and bleomycin (Nippon Kayaku Co Lt, Japan) were dissolved in normal saline.

### Pulmonary fibrosis models and drug administration

Lipopolysaccharide (LPS)-induced model: One hundred male KM mice were randomly divided into the control group, the model group and the NNAV (30, 90, 270 μg/kg) groups. Animals were intragastrically administrated with normal saline or NNAV once daily for 4 days followed by intraperitoneal injection of LPS (Sigma, USA, 5 mg/kg in 0.9% saline water) on the 5^th^ day except the control group. To observe the acute protective effects of NNAV, half of mice in every group were sacrificed 6 h after LPS injection. The rest were subjected to intraperitoneal injection of LPS once a week for 8 weeks to induce chronic pulmonary fibrosis. During the chronic induction period, mice were administered with NNAV or saline once a day until to the end of the experiment.

Bleomycin (BLM)-induced model: Similar to the LPS model, 60 rats were randomly divided into five groups. To induce pulmonary fibrosis, rats were anesthetized with intraperitoneal injection of 4% chloral hydrate (Sigma, Shanghai, China). BLM solution (Nippon Kayaku Co Ltd, Tokyo, Japan, 5 mg/kg in 0.9% saline water) was intratracheally instilled into all the rats except those in control group [5]. Rats in control group were intratracheally administered the same volume of 0.9% saline. NNAV (30, 90, 270 μg/kg) was given orally once a day for 4 days before BLM administration and then was continued for 8 weeks until to the end of the study. All rats were killed 8 weeks after bleomycin exposure.

### Assay of MDA, SOD and GSH

MDA, SOD and GSH in serum and lung tissues were determined with kits following the manufacturer’s instructions (Nanjing Jiancheng Bioengineering Institute, China).

### Arterial blood gas analysis (BGA)

Arterial blood was collected from abdominal aortic artery after 8 weeks NNAV administration. The blood samples were sealed and immediately sent to a local hospital for analysis (No.100 Veterans Hospital, Suzhou, China). BGA was used to evaluate the impairment of respiratory function. The blood power of hydrogen (pH), partial pressure of carbon dioxide (PCO_2_), partial pressure of oxygen (PO_2_), oxygen saturation (SO_2_) and the levels of lactic acid were measured by blood gas analyzer (Radiometer medical equipment Co. Ltd, Copenhagen, Denmark).

### ELISA

Serum IL-1β and TNF-α were determined with ELISA kits according to manufacturer’s protocol (Hushang Bio Technology Co Ltd, Shanghai, China). Samples were measured with a Bio-Rad Benchmark microplate reader at a wavelength of 450 nm. The concentrations of IL-1β and TNF-α were derived from the respective standard curves.

### Assay of hydroxyproline (HYP) content

Pulmonary collagen contents were analyzed with a kit (Nanjing Jiancheng Bioengineering Institute, Nanjing, China) 8 weeks after application of LPS or BLM. The lung tissue was hydrolyzed with NaOH at 95–100°C for 20 min. After neutralization with HCl, the hydrolyzates were diluted with distilled water. Hydroxyproline content in the tissue was assessed with a spectrophotometer at 550 nm in the presence of dimethylaminobenzaldehyde. The results were calculated as μg of hydroxyproline per gram of wet lung tissue [14].

### Histopathological examination

Lung was fixed in 10% buffered formalin solution, embedded in paraffin, sectioned at 5 μm and subjected to hematoxylin-eosin (HE) and Masson’s trichrome staining to detect inflammation or collagen deposition.

### Western blot analysis

Lung was rinsed with saline to remove blood. The lung was then homogenized and centrifuged at 10,000 rpm for 5 min. Thereafter, the supernatant was collected and protein concentrations were determined with the BCA Protein Assay Reagent (Pierce, Rockford, IL, USA). Proteins were resolved with sodium dodecyl sulfate polyacrylamide gel electrophoresis (SDS-PAGE) on a 10% gel. After electrophoresis, proteins were immediately transferred to a nitrocellulose membrane (Bio-Rad Laboratories, Hercules, CA, USA) at 30 mA for 2 h. The membranes were blocked with 5% nonfat dry milk in TBS/Tween (25 mM Tris–HCl, 0.14 M NaCl, 2% Tween 20) at 4°C overnight. Membranes were incubated with primary antibody for one h and the antibody dilutions were as follows: anti-TGF-β1, 1:1000; anti-Smad7, 1:500; anti-p-Smad2/3, 1:500; anti-Collagen I, 1:1000. Washing between and after antibody incubation steps was performed three times for 10 min each with TBS/Tween buffer. Then, the membrane was incubated with fluorescent secondary antibody and scanned with Odyssey^®^ Western Blot Analysis system (LI-COR, Lincoln, NE, USA). The signal intensity of primary antibody binding was quantitatively analyzed with Sigma Scan Pro 5 and was normalized to the loading control β-actin.

### Immunofluorescence analysis

Lung tissues in acute phase of LPS-induced mice was fixed in 10% buffered formalin solution, embedded in paraffin, sectioned at 5 μm thickness and deparaffinized and hydrated using a xylene solution and graded ethanol. After antigen retrieval, sections were rinsed in deionized water. Next, the sections were incubated in 5% bovine serum albumin in phosphate-buffered saline (PBS) for 60 min at room temperature to reduce nonspecific binding of antibody. Sections were then rinsed three times with 1% PBS, covered with primary antibody against NF-κB (NF-κB, 1:100, Cell signaling technology, USA), and incubated in a humidity chamber at 37°C for 60 min. After rinsing three times with 1% PBS, secondary antibodies were added to the sections and incubated in the humidity chamber at 37°C for 30 min. Sections were rinsed, added with DAPI in a humidity chamber at 37°C for 15 min. Sections were rinsed with deionized water, dehydrated with graded ethanol and sealed with neutral resins. Sections were examined with a confocal laser scanning microscope.

### Statistical analysis

All data were presented as mean ± SD, and SPSS16.0 software (SPSS, Inc., USA) was used for statistical analysis. Statistical significance was defined at P <0.05.

## Results

### Effects of NNAV on pulmonary index and hydroxyproline (HYP) content

**Figure 1:**
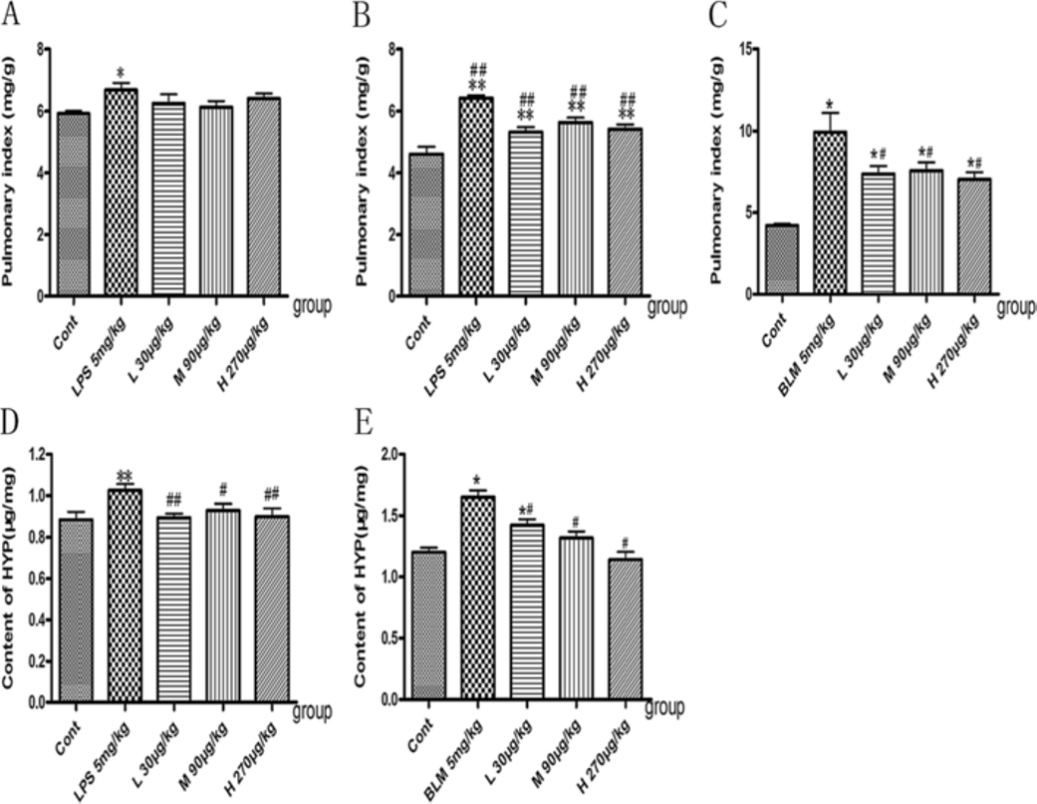
**Effects of NNAV on pulmonary index and HYP content.** Pulmonary fibrosis was induced with intraperitoneal injection of LPS once a week for 8 weeks or a single intratracheal administration of bleomycin. Mice and rats were administered saline or NNAV orally 4 days prior to LPS and BLM and continued until to the end of experiment. **(A)** Pulmonary index 6 h after LPS treatment. **(B)** Pulmonary index 8 weeks after LPS treatment. **(C)** Pulmonary index 8 weeks after BLM treatment. **(D)** HYP levels 8 weeks after LPS treatment. **(E)** HYP levels 8 weeks after LPS treatment. *P<0.05, **P<0.001, model group compared to control group; #P<0.05, ##P<0.001, NNAV-treated groups compared to model group. N =10 per group.

Compared to the control group, pulmonary index (lung weight/ body weight ratio) significantly increased in the LPS- and BLM-induced pulmonary fibrosis models (Figure 1A, B and C; P<0.05). NNAV treatment had no significant effect on pulmonary index 6 h after LPS injection (Figure 1A), but markedly decreased it 8 weeks after LPS, Figure 1B; P<0.05). Similarly, NNAV also significantly reduced the pulmonary index in BLM-induced model (Figure 1C; P<0.05).

Hydroxyproline (HYP) is the main component in extracellular collagen, which is the hallmark of pulmonary fibrosis [4]. In this study, we observed that both LPS-induced (chronic phase) and BLM-induced pulmonary fibrosis models had higher HYP levels than that in the control groups (Figure 1D, E), while NNAV treatment significantly attenuated the elevations in hydroxyproline levels in lung tissues (Figure 1D, E; P<0.05).

### Effects of NNAV on blood gas analysis (BGA) in BLM-induced pulmonary fibrosis

**Figure 2:**
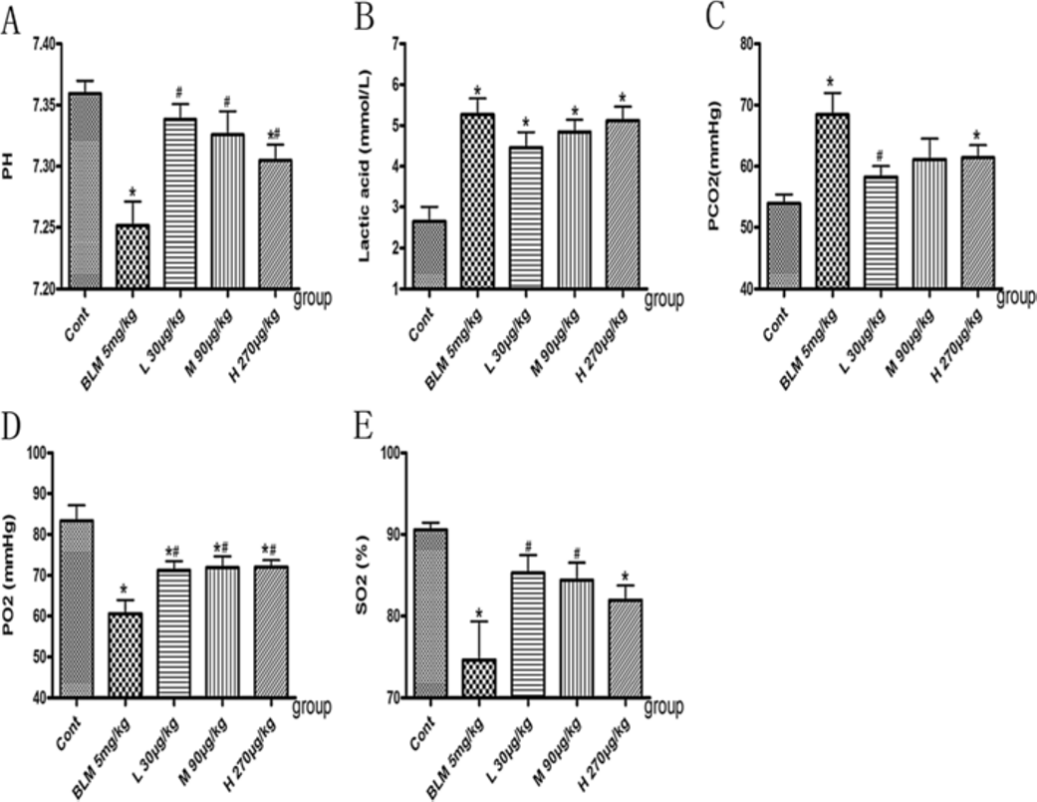
**Arterial blood gas analysis in BLM-induced pulmonary fibrosis.** Rats were administered saline or NNAV orally 4 days prior to BLM and continued until to the end of experiment. Arterial blood was collected in the syringe and sealed. Key indicators of blood gas testing including power of hydrogen (pH, **A**), lactic acid **(B)**, partial pressure of carbon dioxide (PCO2, **C**), partial pressure of oxygen (PO2, **D**), oxygen saturation (SO2, **E**) were measured. *P<0.05, BLM group compared to control group; #P<0.05, NNAV-treated groups compared to BLM group. N =10 per group.

To evaluate the changes in lung function in pulmonary fibrosis, we analyzed the arterial blood gas using acid–base status parameters: power of hydrogen (pH), lactic acid and gas exchange parameters: partial pressure of carbon dioxide (PCO2), partial pressure of oxygen (PO2) and oxygen saturation (SO2). The data showed that rats treated with BLM had a lower value of blood pH (Figure 2A) and a higher level of lactic acid (Figure 2B) than that in control group (*p*<0.05). PCO2 value in the model group was markedly increased (from the baseline of 53.88±5.29 to 68.43±12.28 mmHg, *p*<0.05), while both PO2 value (Figure 2D) and SO2 value (Figure 2E) were significantly decreased (from 83.33±13.13 to 60.57±11.69 mmHg for PO2 value and from 90.53±3.13 to 74.63±16.36 percent for SO2 value, *p*<0.05). NNAV treatment significantly elevated the value of blood pH and reduced lactic acid content (*p*<0.05 vs control). Consistent with acid–base status parameters, low dose, middle dose and high dose of NNAV all significantly increased PO2 and SO2 values and decreased PCO2 value (Figure 2C, D and E).

### Effects of NNAV on the LPS- and BLM-induced histopathological changes in the lungs

**Figure 3:**
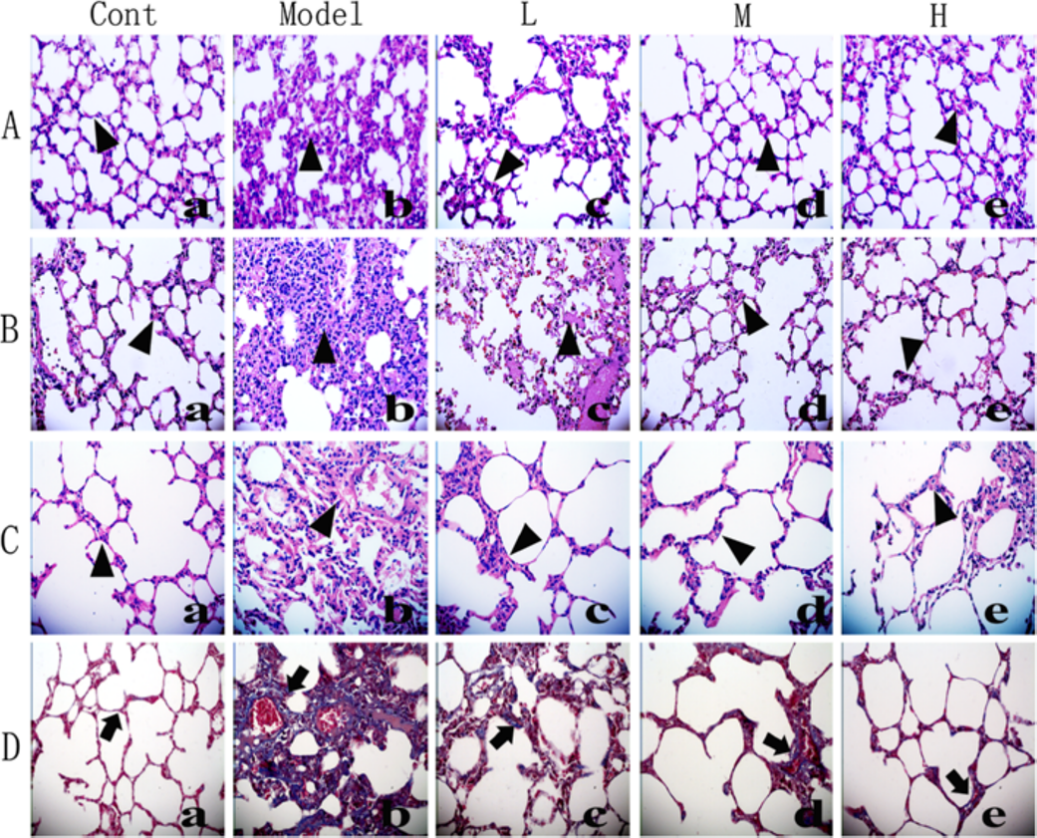
**Effects of NNAV on the histopathological changes in the lung tissue (HE staining and Masson’s trichrome staining, ×400).** Animals were treated as described in the legend to Figure 1. Lung tissue was fixed in 10% formalin, sectioned and stained with HE and trichrome. Inflammatory cell infiltration (▲) and the blue color collagen deposition (↑) in the lung tissue were found. HE staining: **(A)** Six h after LPS. (a) Control. (b) LPS. (c) LPS + NNAV 30 μg/kg. (d) LPS + NNAV 90 μg/kg. (e) LPS + NNAV 270 μg/kg. **(B)** Eight weeks after LPS. (a) Control. (b) LPS-treated lung. (c) LPS + NNAV 30 μg/kg. (d) LPS + NNAV 90 μg/kg. (e) LPS + NNAV 270 μg/kg. **(C)** Eight week after BLM. (a) Control. (b) BLM. (c) BLM + NNAV 30 μg/kg. (d) BLM + NNAV 90 μg/kg. (e) BLM + NNAV 270 μg/kg. **(D)** Masson’s trichrome staining: D(a) Control. D(b) BLM. D(c) BLM + NNAV 30 μg/kg.D(d) BLM + NNAV 90 μg/kg. D(e) BLM + NNAV 270 μg/kg.

Using the HE staining and Masson staining, we found that lung tissue sections from the control group displayed normal structure and no inflammatory or fibrotic lesions (Figure 3A(a), B(a), C(a), D(a)), while a few light blue collagen deposition was observed in the alveolar septa in Masson stained section. In LPS-treated groups (acute phase and long-term) and BLM-treated group, the alveolar septa were significantly thickened (Figure 3A(b), B(b), C(b)), accompanied by infiltration of inflammatory cells. In addition, the blue collagen deposition was markedly increased in the alveolar septa (Figure 3D(b). NNAV treatment (Figure 3A(d, e), B(d, e), C(d, e)) at middle and high dose markedly reduced the infiltration of inflammatory cells, accumulation of collagen deposition and reduced the wall thickness of alveolar septa (Figure 3D(c, d, e)).

### Effects of NNAV on serum levels of IL-1β and TNF-α

**Figure 4:**
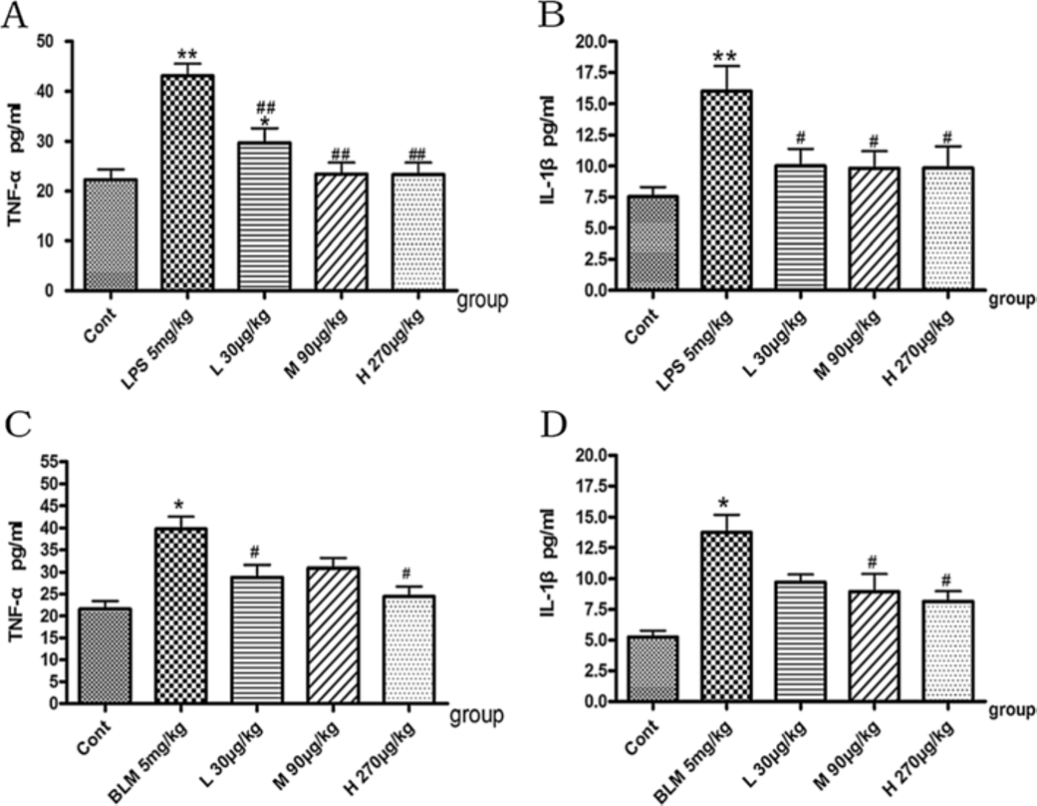
**Effects of NNAV on serum levels of IL-1β and TNF-α.** Animals were treated as described in the legend to Figure 1. The blood was collected from abdomen artery and serum IL-1β and TNF-α were measured with ELISA. **(A)** TNF-α in acute phase of LPS-treated. **(B)** IL-1β in acute phase of LPS-treated. **(C)** TNF-α of BLM-treated. **(D)** IL-1β of BLM-treated *P<0.05, BLM group compared to control group; #P<0.05, NNAV treated groups compared to BLM alone group. N =4 per group.

To determine whether NNAV rendered the protection by inhibiting inflammatory process, we analyzed the serum inflammatory cytokines IL-1β and TNF-α in LPS-induced (acute phase) and BLM-induced pulmonary fibrosis. The data showed that TNF-α (Figure 4A, C) and IL-1β (Figure 4B, D) dramatically up-regulated not only in the acute phase of LPS-induced model but also 8 weeks later in the BLM-induced pulmonary fibrosis. There was a significant reduction in the levels of these cytokines in NNAV-treated animals compared with that in model groups. These results indicated that NNAV attenuated the pulmonary fibrosis, in part, by its anti-inflammatory effects.

### Effects of NNAV on the activation of NF-κB in LPS-induced lung fibrosis

**Figure 5:**
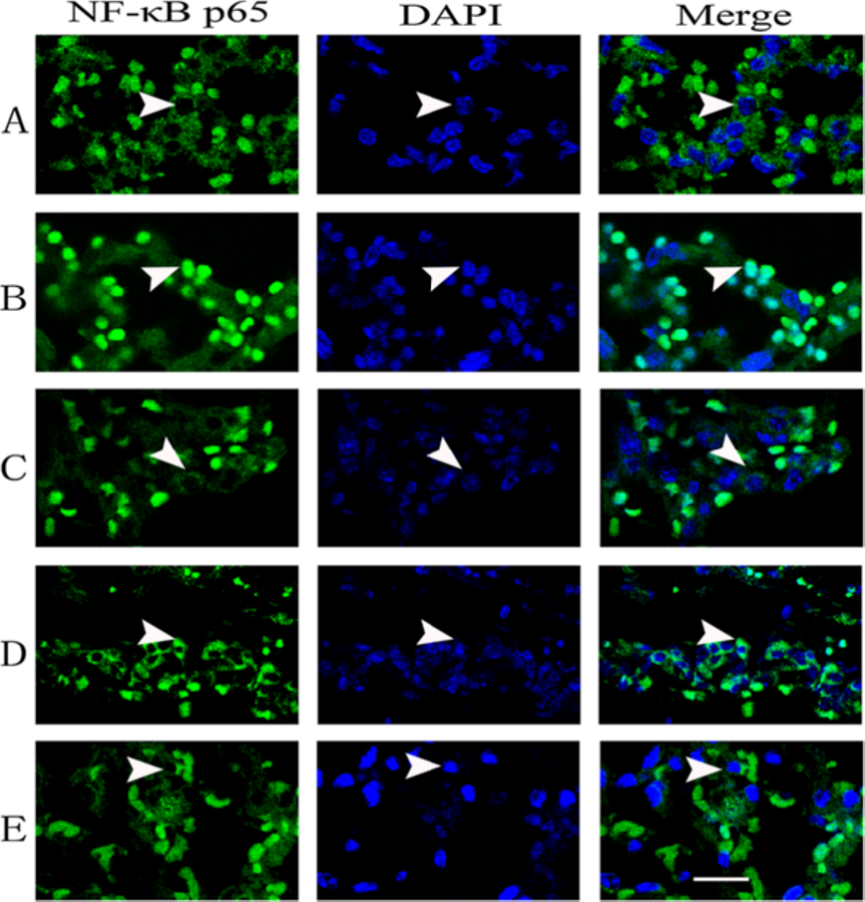
**Effects of NNAV on the nuclear translocation of NF-κB in the lung tissue.** Nuclear translocation of NF-κB induced by LPS injection was detected by confocal microscopy. **(A)** Expression of NF-κB p65 in control group. **(B)** Expression of NF-κB p65 in acute phase of LPS-treated group. **(C)** Expression of NF-κB p65 in Low dose of NNAV-treated group (30 μg/kg). **(D)** Expression of NF-κB p65 in Middle dose of NNAV-treated group (90 μg/kg). **(E)** Expression of NF-κB p65 in High dose of NNAV-treated group (270 μg/kg). Scale bar equals 20 μm. See text for details.

The transcription factor NF-κB plays a key role in immune response and lung fibrosis. To determine the molecular mechanisms of NNAV’s anti-inflammatory effects, we explored the effects of NNAV on the activation of NF-κB with immunofluorescence. Data showed that p65 was distributed mainly in the cytoplasm in control lungs, while it rapidly translocated into the nucleus when mice were injected with LPS (6 h), which denoted the activation of NF-κB. NNAV (90 and 270 μg/kg) significantly reduced the translocation of p65 from the cytoplasm to the nucleus (Figure 5). These results indicated that the inhibition of NF-κB was involved in the protection of NNAV on the pulmonary fibrosis.

### Effects of NNAV on TGF-β/Smad pathway in the lung tissue

**Figure 6:**
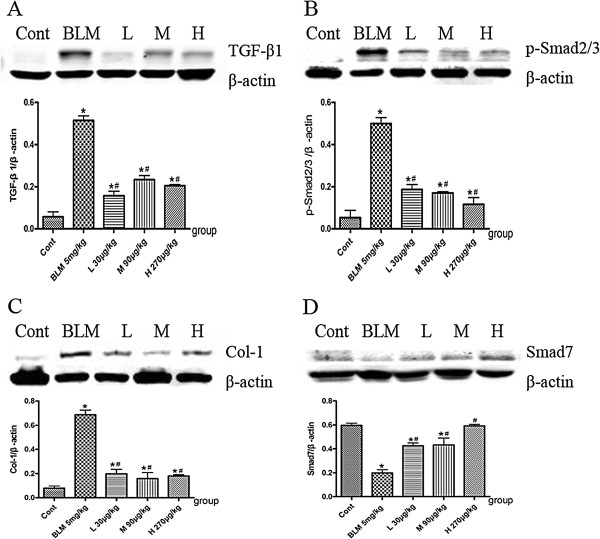
**Effects of NNAV on TGF-β/Smad pathway in the lung tissue.** Animals were treated as described in the legend to Figure 1. All rats were killed 8 weeks after bleomycin exposure. The Lung was collected, homogenized and centrifuged, and then the supernatant was collected for Western blot analysis. **(A)** Protein expression of TGF-β1. **(B)** Protein expression of p-Smad2/3. **(C)** Protein expression of Collagen I. **(D)** Protein expression of Smad7. Bars represent the mean ± SD of a minimum of N=6 per group. *P<0.05, BLM group compared to control group, #P<0.05, NNAV-treated groups compared to BLM group.

It is well accepted that TGF-β is a key cytokine in the process of fibrogenesis via the intracellular signaling pathway involving Smad2 and Smad3. In order to determine the role of the TGF-β/Smad pathway in NNAV-mediated anti-inflammation and anti-fibrosis effects, we detected the protein expression of TGF-β1, p-Smad2/3, collagen I (a marker of fibroblast activation) and Smad7 (a negative regulative protein by preventing phosphorylation of Smad2/3) [15–17]. We found that BLM-treated model group had a dramatic increase in the levels of TGF-β1, p-Smad2/3 and collagen I in lung tissue, accompanied by a significant decrease in the levels of Smad7 (Figure 6). NNAV treatment caused a significant decrease in TGF-β1, phosphorylated Smad2/3 and type I collagen levels (Figure 6 A, B and C, *p*<0.05) concomitant with an elevation in Smad7 levels (Figure 6D, *p*<0.05). These results indicate that NNAV can inhibit the TGF-β/Smad signaling pathway.

### Effects of NNAV on oxidative stress

**Figure 7:**
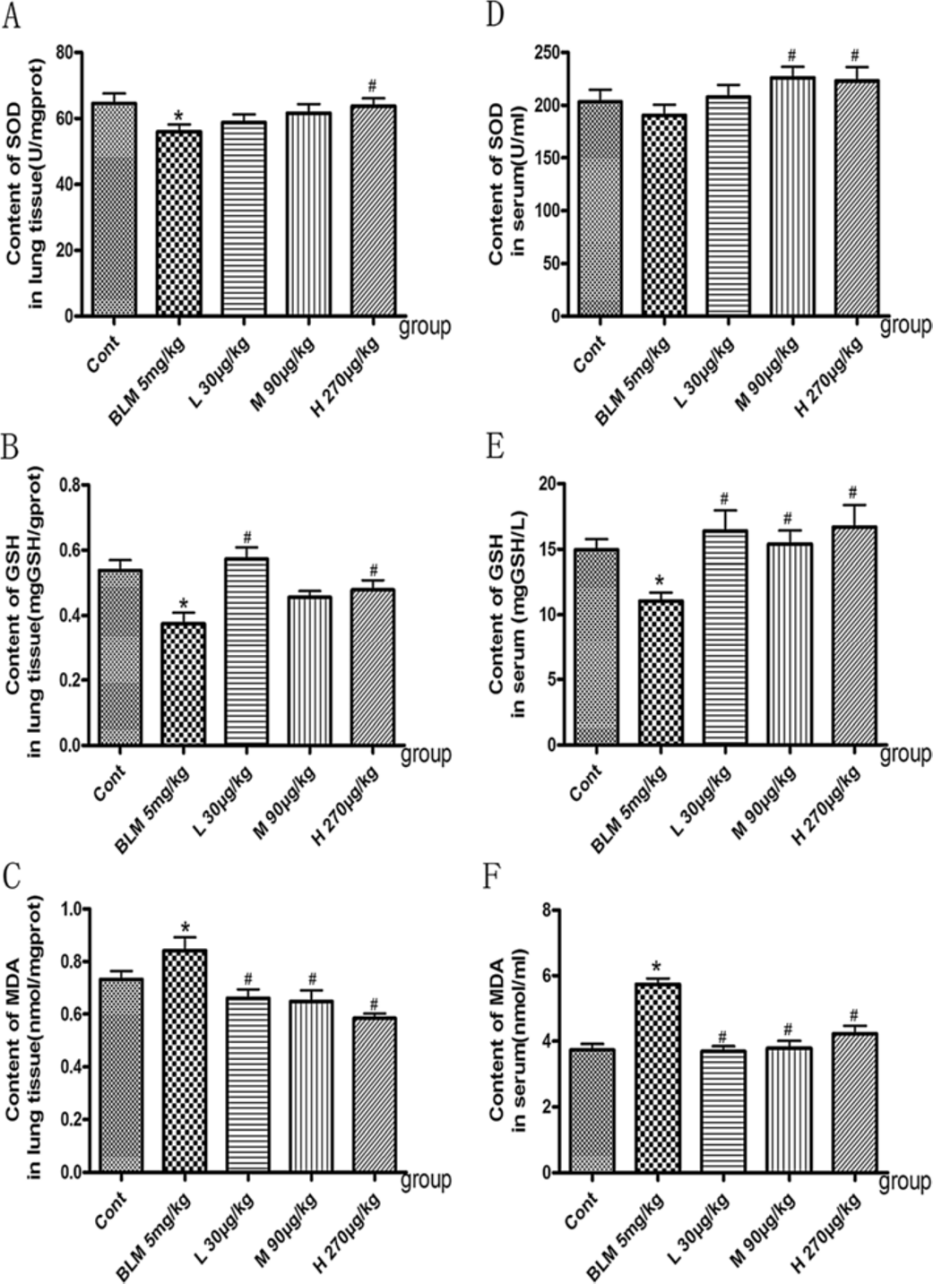
**Effects of NNAV on the levels of MDA, SOD and GSH.** Animals were treated as described in the legend to Figure 1. The blood was collected from abdomen artery and SOD, GSH and MDA were measured in the serum and lung tissue. **(A)** SOD in the lung tissue. **(B)** GSH in the lung tissue. **(C)** MDA in the lung tissue. **(D)** SOD in the serum. **(E)** GSH in the serum. **(F)** MDA in the serum. *P<0.05, compared to control group, #P<0.05, NNAV-treated groups compared to BLM alone group. N =10 per group.

Oxidative stress is one of prominent mechanisms involved in the pathogenesis of pulmonary fibrosis [18, 19]. To explore whether NNAV’s protection was related to anti-oxidative effect, we determined the levels of SOD, GSH and MDA in lung tissue and in serum. The results showed that BLM decreased the levels of SOD (Figure 7A, D) and GSH (Figure 7B, E), companied with an increased level of MDA (Figure 7C, F). NNAV treatment elevated the levels of SOD and GSH and reduced the levels of MDA both in lung tissue and in serum (Figure 7, *P*<0.05). These results suggested that NNAV could attenuate the BLM-induced oxidative stress.

## Discussion

In this study, we present experimental evidence demonstrating the protective effects of NNAV on pulmonary fibrosis. NNAV not only ameliorated histopathological impairment in murine lung fibrosis models but also improved the lung gas-exchange function. The first model is LPS-induced mouse lung fibrosis which is inflammation related [20]. We assessed the effects of NNAV on acute phase (6 h) and chronic phase (8 weeks) in LPS-induced lung inflammatory injury. Consistent with the report, LPS injection resulted in considerable lung tissue injury in a few hours, which is characterized by an increased level of pro-inflammatory cytokines, neutrophil accumulation in the alveolar and interstitial space and alveolar wall thickening. NNAV markedly attenuated these inflammatory responses. To further validate the protection, we observed the effects of NNAV in the chronic phase of this model. When LPS administration continued for 8 weeks, the lung tissue had increased levels of hydroxyproline (HYP) content, collagen I and thickness of alveolar septa, suggesting pulmonary fibrosis was produced. NNAV dramatically decreased the HYP content and alleviated the lung tissue fibrosis.

After a poisonous snake bite, in which the venom is delivered to the veins and tissues in its native form, severe inflammation is induced. In our study, we administered NNAV orally and some components may be destroyed by protease. The therapeutic dose of NNAV used in the study was considerable small compared to a dose received by snake bite. In addition, a previous study by Zhu et al. [10] showed that high temperature, 100°C, cause the increased expression of low molecular weight proteins, such as cardiotoxin and neurotoxin. All these might attribute to its different effects of NNAV on inflammation from a snake bite.

The second model is BLM-induced rat pulmonary fibrosis. BLM is used as a chemotherapeutic agent for the treatment of human cancer. Its common adverse effect is pulmonary fibrosis. The intratracheal administration of BLM can cause pulmonary fibrosis and is a commonly used animal model for pulmonary fibrosis [21, 22]. The present results showed that BLM administration caused the robust histopathological changes of the lung, including thickness of alveolar septa, smaller alveolar space, infiltration of inflammatory cells and the accumulation of collagen deposition. NNAV treatment again markedly reduced the accumulation of collagen deposition, decreased hydroxyproline content and meliorated the wall thickness of alveolar septa. BGA remains a first-step diagnostic approach in patients with suspected lower pulmonary function [23, 24]. The current results indicated that BLM-treated rats had decreased values of pH, PO2 and SO2 and increased levels of lactic acid and PCO2 in contrast to control group. We found that NNAV treatment improved lung gas exchange function.

Cellular inflammation is the pathologic hallmark in the lung parenchyma of patients with pulmonary fibrosis [3]. Actually the majority of animal models of fibrosis including the above described models start with inflammation [22]. TNF-α is a key cytokine in pulmonary fibrosis, which induces expression of adhesion molecule, recruitment of inflammatory cells into the lungs and synthesis of other cytokines [3, 4]. In our study, both LPS- and BLM-induced models are characterized by the increased levels of TNF and IL-1β in serum. NNAV significantly reduced the increases in TNF-α and IL-1β levels in both models. Therefore, these results suggest that NNAV might produce the protection on lung fibrosis by anti-inflammatory effect. It is generally accepted that the regulation of pro-inflammatory cytokines is mediated, at least partly, by NF-κB. Therefore, NF-κB can be taken as a potential target for the therapy of lung fibrosis. Since LPS is the typical activator of NF-κB pathway, we observed the effects of NNAV in the acute phase of LPS-induced pulmonary injury model. NNAV decreased the LPS-induced the nuclear translocation of NF-κB. This provides one possible mechanism of for NNAV’s inhibitory effect on pro-inflammatory cytokines.

Transforming growth factor *β* (TGF-*β*) is implicated in the initiation and progression of fibrosis [3]. The profibrotic effects of TGF-*β* are mediated by its multiple actions, including induction of myofibroblasts, increase of matrix synthesis, and inhibition of collagen breakdown. Most of these effects are thought to be mediated through the Smad signaling pathway. Once activated, TGF-*β* signals through transmembrane receptors that trigger phosphorylation of Smad2/3 protein, which modulates transcription of important target genes, including pro-collagen I and III. The phosphorylation of Smad2/3 can be inhibited by Smad7 [15]. It has been established that alveolar macrophages stimulated by BLM secrete large quantities of biologically active TGF-*β*. Our results demonstrated that NNAV lowered the total and biologically active TGF-β levels in the lung tissue compared with BLM-treated model group. There was also a significant difference in the levels of phosphorylation of Smad2/3 and Smad7 between the BLM-treated model group and NNAV-treated groups. The expression of collagen I was markedly upregulated by BLM. NNAV greatly reduced the expression of collagen I. These suggest that NNAV inhibits the TGF-*β*-mediated transcription of profibrotic genes downstream of the phosphorylation and nuclear translocation of Smads.

Oxidant stress is a key player in the pathogenesis of pulmonary fibrosis [18, 19]. Both patients and experimental models of lung fibrosis have displayed marked elevation of oxidant burden and disturbed antioxidant/oxidant balance [3]. Several studies also suggest that reactive oxygen species can cause activation of growth-regulatory cytokines, including TGF-*β*. In light of the remarkable free radical scavenging activity of some fractions of NNAV, in this study we demonstrated that NNAV did affect antioxidant/oxidant balance in BLM-induced lung fibrosis. The evidence is that NNAV treatment elevated the levels of SOD and GSH but reduced level of MDA both in lung tissue and in serum.

Some snake toxins have anticoagulant effects. Mizuno et al. [25] described factor X-binding protein (X-bp), an anticoagulant protein, from snake venom. And one of the components of Naja nigricollis venom, phospholipase A2 (PLA2), has also been shown to be an anticoagulant [26]. In addition, treatment of pulmonary fibrosis patients with heparin and warfarin (anticoagulants) reduces the coagulation that occurs in the lungs as a result of vascular injury, and endothelial disruption [27]. Whether NNAV ameliorated pulmonary fibrosis partly through anti-coagulation action needs to be addressed in the future.

## Conclusions

In summary, the present study demonstrated that NNAV inhibited LPS- and BLM-induced pulmonary fibrosis and improved gas-exchange function. This protection is associated with anti-inflammatory and anti-oxidative capability of NNAV. The study suggests a potential therapeutic usefulness of NNAV on pulmonary fibrosis.

## Abbreviations

NNAV: Naja naja atra venom
HYP: Hydroxyproline
LPS: Lipopolysaccharide
BLM: Bleomycin
HE: Hematoxylin-eosin
BGA: Arterial blood gas analysis
TGF-β: Transforming growth factor β.
